# Three novel *Pseudomonas* phages isolated from composting provide insights into the evolution and diversity of tailed phages

**DOI:** 10.1186/s12864-017-3729-z

**Published:** 2017-05-04

**Authors:** Deyvid Amgarten, Layla Farage Martins, Karen Cristina Lombardi, Luciana Principal Antunes, Ana Paula Silva de Souza, Gianlucca Gonçalves Nicastro, Elliott Watanabe Kitajima, Ronaldo Bento Quaggio, Chris Upton, João Carlos Setubal, Aline Maria da Silva

**Affiliations:** 1 Departamento de Bioquímica, Instituto de Química, Universidade de São Paulo, São Paulo, Brazil; 2 Programa de Pós-Graduação Interunidades em Bioinformática, Universidade de São Paulo, São Paulo, Brazil; 3 Departamento de Fitopatologia e Nematologia, Escola Superior de Agricultura Luiz de Queiroz, Universidade de São Paulo, Piracicaba, Brazil; 4 Biochemistry and Microbiology, University of Victoria, Victoria, BC Canada; 5 Biocomplexity Institute of Virginia Tech, Blacksburg, VA USA

**Keywords:** Bacteriophages, Composting, Homing endonucleases, tRNA genes, Genomics, Metagenomics, *Pseudomonas aeruginosa*, *Siphoviridae*, *Podoviridae*

## Abstract

**Background:**

Among viruses, bacteriophages are a group of special interest due to their capacity of infecting bacteria that are important for biotechnology and human health. Composting is a microbial-driven process in which complex organic matter is converted into humus-like substances. In thermophilic composting, the degradation activity is carried out primarily by bacteria and little is known about the presence and role of bacteriophages in this process.

**Results:**

Using *Pseudomonas aeruginosa* as host, we isolated three new phages from a composting operation at the Sao Paulo Zoo Park (Brazil). One of the isolated phages is similar to *Pseudomonas* phage Ab18 and belongs to the *Siphoviridae YuA-like* viral genus. The other two isolated phages are similar to each other and present genomes sharing low similarity with phage genomes in public databases; we therefore hypothesize that they belong to a new genus in the *Podoviridae* family. Detailed genomic descriptions and comparisons of the three phages are presented, as well as two new clusters of phage genomes in the Viral Orthologous Clusters database of large DNA viruses. We found sequences encoding homing endonucleases that disrupt a putative ribonucleotide reductase gene and an RNA polymerase subunit 2 gene in two of the phages. These findings provide insights about the evolution of two-subunits RNA polymerases and the possible role of homing endonucleases in this process. Infection tests on 30 different strains of bacteria reveal a narrow host range for the three phages, restricted to *P. aeruginosa* PA14 and three other *P. aeruginosa* clinical isolates. Biofilm dissolution assays suggest that these phages could be promising antimicrobial agents against *P. aeruginosa* PA14 infections. Analyses on composting metagenomic and metatranscriptomic data indicate association between abundance variations in both phage and host populations in the environment.

**Conclusion:**

The results about the newly discovered and described phages contribute to the understanding of tailed bacteriophage diversity, evolution, and role in the complex composting environment.

**Electronic supplementary material:**

The online version of this article (doi:10.1186/s12864-017-3729-z) contains supplementary material, which is available to authorized users.

## Background

Viruses present remarkable diversity regarding morphology, genomes, and proteins [1]. Among viruses, bacteriophages (or simply phages) are a group of special interest, given their interactions with bacteria that are important for biotechnology and human health. As new phage genomes are characterized, unusual features are found, including new genes and novel genome architectures [2–4]. Thus, the study of phage diversity can contribute to the understanding of their evolution and their influence on any microbial community.

Composting is a diverse microbial environment in which complex organic molecules such as lignocellulose are converted into humus-like substances suitable for use as a soil amendment [5]. The study of composting microbial communities is important for elucidating the pathways of biomass degradation, and has contributed to the discovery of novel microorganisms and valuable enzymes for biotechnological applications [6, 7]. Aside from the great diversity of bacteria and fungi species in this environment [8–11], phages have also been identified in composting material [12–14]. Recent studies have reported novel phage genomes in composting [13] and interesting features, such as phage thermostable enzymes [14].

In this work, we describe three new phages isolated from a composting operation at the Sao Paulo Zoo Park (Brazil) using *Pseudomonas aeruginosa* PA14 as host. This reference strain is a clinical and highly virulent isolate that represents the most common clonal group worldwide [15]. Along with the characterization of these phages, this work also presents results concerning transcribed phage genes and phage abundance variation from a three-month time-series sampling of the composting process. To our knowledge, this is the first study to present such results concerning the complex microbial context in which these phages live.

## Results and Discussion

### Phage isolation and sequencing

We screened composting samples from the São Paulo Zoo Park (São Paulo, Brazil) [7] for phages infecting *Pseudomonas aeruginosa* PA14, in order to access a slice of the cultivable phage diversity in this complex microbial community. Three new phages were isolated, which we named *Pseudomonas* phage ZC01, ZC03 and ZC08. Their genomes were fully sequenced, assembled, and annotated. Overall characteristics of these phage genomes are summarized in Table 1. A final genome of 57,061 bp was obtained for phage ZC01, which was linearized following phage YuA reference and close genomes [16]. Assemblies for isolates ZC03 and ZC08 resulted in two slightly different sequences with 69,844 bp and 70,774 bp, respectively. For all three phage genomes, coverage was above 8000x and uniform through the entire contig. Metrics about the assembly process are available in the Additional file 1: Table S1.

**Table 1:** New phages genomic features

Feature	Phage ZC01	Phage ZC03	Phage ZC08
Accession Number	KU356689	KU356690	KU356691
Genome size (bp)	57,061	69,844	70,774
GC content (%)	63	42	42
Genes predicted	78	85	83
tRNA genes	None	10	9 + 1 Pseudogene

Genomic features of the three new *Pseudomonas* phages described in this work

### Genomic and functional characterization of phage ZC01

The majority of the ZC01 genome consists of coding sequences, with the exception of three main non-coding regions: 370 bp at the 5’ end, 249 bp around the 8 kbp position and 933 bp around the 31 kbp position. A common characteristic for these non-coding regions is their lower GC content (41%) compared to the average GC content for the entire ZC01 genome (63%, Table 1). This variation is due to an increase of T nucleotides from a mean value of 16% in the genome to up to 37% in these non-coding regions.

We have searched the National Center for Biotechnology Information (NCBI) nt and microbial RefSeq genomes databases [17] for genomes similar to phage ZC01. The most similar genomes include phage Ab18 (98% coverage and 96% identity), phage PaMx11 (80% coverage and 72% identity) and phage YuA (31% coverage and 69% identity) [16, 18, 19]. Phage YuA belongs to the *YuA-like virus* genus of the *Siphoviridae* family [20]. Based on this information, we have created a new cluster of viral genomes in the Viral Orthologous Clusters database (VOCs) [21], which was named *Siphoviridae YuA-like* and is publicly available through the VOCs Java client [22]. Genomes in the *YuA-like* cluster were selected using BLASTN [23] results and the *Jaccard* index of similarity based on shared genes, as detailed in the Methods section. Clusters created in this study are not meant to reflect strict taxonomy groups, but similarity through shared genes only. This cluster contains 14 different genomes (listed in Table 2), 932 genes and 401 ortholog groups (OGs).

**Table 2:** Phage genomes assigned to *Siphoviridae* YuA-like and *Podoviridae* N4-like VOCs clusters

*Siphoviridae YuA-like* cluster^a^		*Podoviridae N4-like* cluster^a^	
Phage species	Accession number	Phage species	Accession number
*Burkholderia* phage BcepGomr	NC_009447	Enterobacter phage EcP1	NC_019485
Phage phiJL001	NC_006938	Enterobacteria phage N4	NC_008720
*Pseudomonas* phage 73	NC_007806	*Erwinia* phage vB_EamP-S6	NC_019514
*Pseudomonas* phage Ab18	LN610577	*Escherichia* phage vB_EcoP_G7C	NC_15933
*Pseudomonas* phage B3	NC_006548	*Pseudomonas* phage LIT1	NC_013692
*Pseudomonas* phage D3112	NC_005178	*Pseudomonas* phage LUZ7	NC_013691
*Pseudomonas* phage DMS3	NC_008717	*Pseudomonas* phage ZC03^b^	KU356690
*Pseudomonas* phage M6	NC_007809	*Pseudomonas* phage ZC08^b^	KU356691
*Pseudomonas* phage MP22	NC_009818	Roseophage DSS3P2	NC_012697
*Pseudomonas* phage MP29	NC_011611	Roseophage EE36P1	NC_012696
*Pseudomonas* phage MP38	NC_011611		
*Pseudomonas* phage PaMx11	NC_0028770		
*Pseudomonas* phage YuA	NC_010116		
*Pseudomonas* phage ZC01^b^	KU356689		

^a^Clusters created in this work are not meant to reflect strict taxonomy groups as defined by the ICTV. ^b^New phages described in this study. Data is available in the VOCs database of large DNA viruses

All ten most conserved genes in the *YuA-like* cluster have orthologous representatives in phage ZC01 (Table 3). Likewise, conserved DNA helicase, RecD-like protein, deoxyuridylate hydroxymethyltransferase and DNA polymerase A were annotated and assigned to ortholog groups with six or more orthologous genes. Due to the high similarity among phages ZC01, Ab18 and PaMx11, there are 36 ortholog groups shared by them only. These include well-known proteins such as a holin and a DNA ligase, but also several hypothetical proteins. Moreover, phages Ab18 and ZC01 share two specific ortholog groups (VOCs ID: 18968, 18971), which were annotated as hypothetical and have no similarity with anything else in the NCBI nr database. Their function remains to be discovered. In addition, we found in the ZC01 genome a gene coding for a protein that is the only member of the VOCs ortholog group 18954. The predicted protein is the Rz1 smaller lipoprotein. It was manually annotated through the inspection of the Rz larger lipoprotein ORF (Rz1 is nested within Rz). This annotation was based on similar findings for the lambda phage genome, where Rz1 was experimentally isolated and characterized [24]. We verified that ZC01, Ab18 and PaMx11 share orthologous proteins to the Rz larger lipoprotein (VOCs ID: 22052); therefore, we conclude that the Rz1 smaller nested lipoprotein is also encoded in Ab18 and PaMx11 genomes, but it was not annotated (see Additional file 2 for the tblastn alignment of this genomic region).

**Table 3:** List of the 20 most conserved ortholog groups in the *Siphoviridae* YuA-like cluster

VOCs ID^a^	Ortholog Group	Number of genes	Number of genomes
18328	Putative tail assembly protein (PSP-YuA-073)	12	12
18331	Putative tail protein (PSP-YuA-076)	12	12
18325	Tail fiber protein (PSP-YuA-070)	12	11
18326	Structural phage protein (PSP-YuA-071)	11	11
18327	Tail assembly protein (PSP-YuA-072)	11	11
18330	Conserved tail assembly protein (PSP-YuA-075)	11	11
18306	Terminase large subunit (PSP-YuA-051)	8	8
18323	Virion structural protein (PSP-YuA-068)	8	8
18278	Putative deoxycytidylate deaminase (PSP-YuA-023)	7	7
18318	Structural phage protein (PSP-YuA-063)	7	7
18257	DNA helicase (PSP-YuA-002)	6	6
18258	Hypothetical protein (PSP-YuA-003)	6	6
18259	Hypothetical protein (PSP-YuA-004)	6	6
18260	Hypothetical protein (PSP-YuA-005)	6	6
18261	RecD-like DNA helicase (PSP-YuA-006)	6	6
18269	Hypothetical protein (PSP-YuA-014)	6	6
18272	Deoxyuridylate hydroxymethyltransferase (PSP-YuA-017)	6	6
18273	Hypothetical protein (PSP-YuA-018)	6	6
18275	Bacteriophage conserved protein (PSP-YuA-020)	6	6
18276	DNA Polymerase I (PSP-YuA-021)	6	6

^a^Data is available in the VOCs database of large DNA viruses

**Fig. 1:**
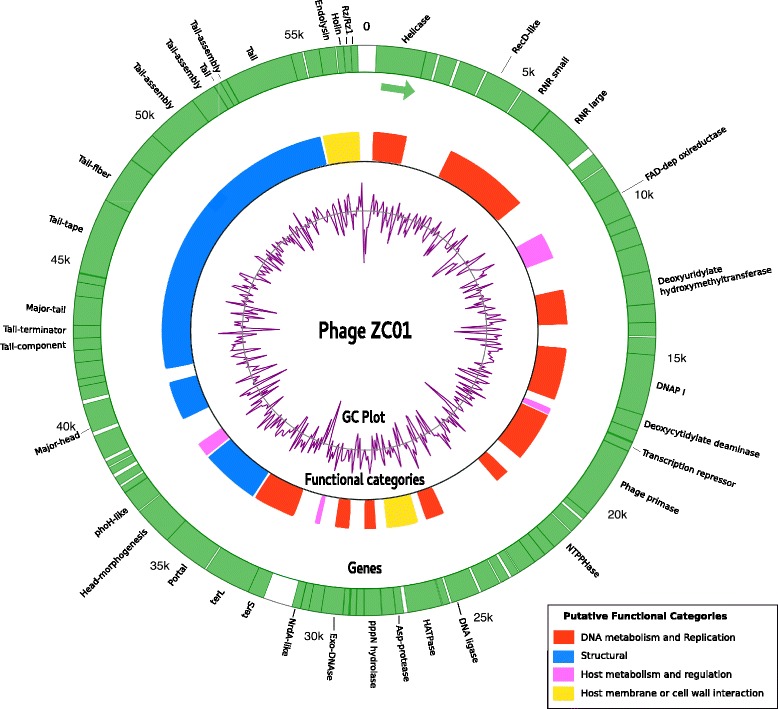
Phage ZC01 genome plot. Circular representation of the *Pseudomonas* phage ZC01 genome. The outer circle represents genes (all genes are on the plus strand, as indicated by the arrow). Putative functional categories were defined according to annotation and are represented by colors. Gaps in the functional block circle represent proteins with unknown function. The central graph (in purple) shows genomic GC content variation computed in 100 bp windows

Functional annotation of the predicted proteins based on VOCs clusters context and additional tools showed that 41% of ZC01 proteins have unknown function. “DNA metabolism and replication” and “structural proteins” are the functional categories with most genes. Few protein products were annotated as involved in host interaction pathways, e.g., membrane or cell wall interaction and metabolism regulation. ZC01 genome annotation is summarized in Fig. 1. Detailed annotation of the ZC01 genome and genes is presented as Additional file 3: Table S2.

### Genomic and functional characterization of phages ZC03 and ZC08

Phages ZC03 and ZC08 present very similar genomes, with 95% of their nucleotide sequences aligned averaging 98% identity. Differences are mainly due to two indels at the 47 kbp and 51 kbp positions. The first indel consist of a unique sequence (~800 bp) present in phage ZC03, while the second indel consists of a unique sequence (~1000 bp) present in phage ZC08.

ZC03 and ZC08 non-coding regions also present variation on the GC content. However, in contrast to ZC01, the ZC03 and ZC08 non-coding regions display an increase in the GC content (~60%) with respect to the average CG content of the genome (42%, Table 1). The average GC content from phages ZC03 and ZC08 significantly diverges from the GC content of the assumed host genome (66%), suggesting that *P. aeruginosa* may not be the optimal host for these two phages [25].

BLASTN searches of ZC03 and ZC08 genomes against the NCBI RefSeq database returned hits with low genomic coverage and identity. The best hits included Enterobacteria phage N4 (10% coverage and 70% identity), *Erwinia* phage Ea9-2 (8% coverage and 70% identity) and Enterobacteria phage IME11 (7% coverage and 71% identity). These results strongly indicate that phages ZC03 and ZC08 are rather different from known phage species and that they probably belong to a new genus in the *Podoviridae* family. For this reason, creating a cluster of similar genomes in this case was challenging due to the shortage of similar genomes. Phage N4 is the most similar known genome and also the only officially representative of a genus recognized by the International Committee on Taxonomy of Viruses (ICTV), the *N4-like-virus* [20]. Notwithstanding, several phages have been reported as strongly related to this genus, including *Pseudomonas*, *Escherichia* and *Achromobacter* phages [26]. Thus, considering BLASTN results and the *Jaccard* index of similarity to select phage genomes, we created a new cluster of viruses in the VOCs database, which we named *Podoviridae N4-like cluster*. Ten different genomes were selected to be part of this cluster (Table 2), comprising 876 genes and 491 ortholog groups. Data for the YuA-like, N4-like and a third model cluster for T4-like myophages are publicly available through the VOCs Java client at the Viral Bioinformatics Resource Center (VBRC) web platform [22].

The ZC03 and ZC08 genomes harbor representatives from all the 15 core genes found for the N4-like cluster, or 14 core genes for the *N4-like* genus according to the literature [27]. These genes include RNA polymerases 1 and 2, DNA helicase, DNA polymerase, primase, exonuclease, terminase small and large subunits and coat proteins (Table 4).

**Table 4:** List of the 15 most conserved ortholog groups in the *Podoviridae* N4-like cluster

VOCs ID^a^	Ortholog Group	Number of genes	Number of genomes
18472	RNAP1 (EBP-N4-015)	10	10
18473	RNAP2 (EBP-N4-016)	10	10
18481	AAA ATPase containing protein (EBP-N4-024)	12	10
18494	DNA helicase (EBP-N4-037)	10	10
18496	DNAP (EBP-N4-039)	10	10
18499	Putative exonuclease (EBP-N4-042)	10	10
18500	Putative primase (EBP-N4-043)	10	10
18501	gp44 (EBP-N4-044)	10	10
18502	Single-stranded DNA-binding protein (EBP-N4-045)	10	10
18512	gp55 (EBP-N4-055)	10	10
18513	Major coat protein (EBP-N4-056)	10	10
18514	gp57 (EBP-N4-057)	10	10
18516	Putative portal protein (EBP-N4-059)	10	10
18525	Terminase large subunit (EBP-N4-068)	10	10
18526	gp69 (EBP-N4-069)	10	10

^a^Data is available in the VOCs database of large DNA viruses

Forty-four genes were assigned to specific ZC03/ZC08 ortholog groups, corresponding to more than half of the full set of genes in each genome. ZC03 and ZC08 genes are very different from gene sequences available in public databases, indicating the high degree of novelty of these genomes. Among these 44 specific genes, only two products could be annotated: a putative peptidoglycan hydrolase gp181 (VOCs ID: 18902) and a putative homing endonuclease (VOCs ID: 18919). ZC03 genome contains six unique genes and an additional tRNA gene that is not present in the ZC08 genome (probably a pseudogene, as we discuss later). On the other hand, ZC08 presents five unique genes that are not present in the ZC03 genome. Given the evidence, we hypothesize that these two phages have diverged in the recent evolutionary past.

**Fig. 2:**
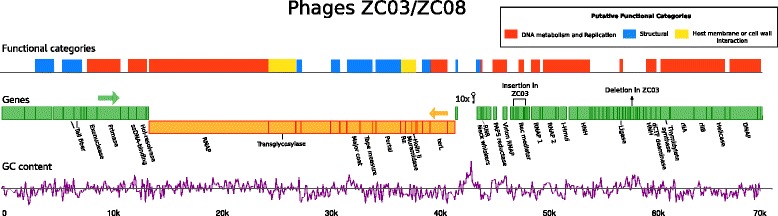
Phage ZC03 genome plot. Linear representation of the *Pseudomonas* phage ZC03 genome. The two central bands represent genes being codified by the plus strand (green) or minus strand (orange). Putative functional categories were defined according to annotation and are represented by colors in the top strand. Gaps in the functional blocks band represent proteins with unknown function. The bottom purple graph shows GC content variation computed in 100 bp windows. Hairpin symbol shows genome region where tRNA genes were predicted. Ins/del regions are shown comparing ZC03 and ZC08 phages

Functional annotation of the predicted proteins for phages ZC03 and ZC08 based on VOCs clusters context and other tools showed that approximately 50% of the predicted proteins have unknown function. ZC03/ZC08 genome annotation and features are summarized in Fig. 2. For a complete list of evidence and annotation, see Additional file 4: Table S3.

### ZC03 and ZC08 specific genes and differences

Given their overall similarity, the differences between the ZC03 and ZC08 genomes can help in the understanding of viral evolution. Major differences include one gene region (ZC03_002 and ZC08_002) with lower nucleotide identity than its neighborhood (48 to 94% identity, respectively) and three indel regions encoding several genes (Table 5).

**Table 5:** Phages ZC03 and ZC08 specific genes

Gene	Protein Size (aa)	Annotation	BLAST and CDD weak hits^a^
ZC03_002	331	Hypothetical protein	No significant hits
ZC03_047	143	Hypothetical protein	Conserved Hypothetical protein (CDD:DUF2461), Cellulase-like domain (pfam12876)
ZC03_051	60	Hypothetical protein	SH3 Proline recognition superfamily (CDD:cl17036)
ZC03_052	61	Hypothetical protein	Metallophosphatase superfamily (CDD:cl13995), Conserved hypothetical protein (CDD:DUF1501)
ZC03_053	166	DrpA-like recombination mediator (InterPro:IPR003488)	---
ZC03_054	32	Hypothetical protein	Flagellar basal body-associated protein FliL (COG1580)
ZC03_055	37	Hypothetical protein	No significant hits found
ZC03_073	65	Hypothetical protein	Metallochaperone hypA superfamily (pfam01155)
---	---	---	---
ZC08_002	337	Hypothetical protein	Tail assembly protein *Xyllela* phage Salvo (AHB12240.1)
ZC08_048	82	Hypothetical protein	*Borrelia* lipoprotein (pfam00820)
ZC08_055	209	Putative HNH homing endonuclease	---
ZC08_073	47	Hypothetical protein	No significant hits found
ZC08_074	132	Hypothetical protein	Phage head-tail joining protein (COG5614)
ZC08_075	129	Hypothetical protein	Metal-responsive transcriptional regulator (pfam15611)

^a^Hits were considered weak hits if the alignment presented coverage >= 0.3 and identity >= 20% through HMM or PSSM searches

The genes ZC03_002 and ZC08_002 were annotated as hypothetical proteins, although BLASTP results show similarity (coverage 41%, identity 29%) with a tail assembly protein of *Xylella* phage Salvo (AHB12240.1). Genes ZC03_002 and ZC08_002 were assigned to separate individual VOCs ortholog groups, likely because their encoded amino acid sequences present only 29% identical residues in a full alignment. Multiple alignment of the genomes indicates a syntenic relationship between these genes, providing additional evidence for the hypothesis that they are distant orthologs.

ZC03 presents a cassette of genes that might have been originated from a horizontal gene transfer event. Five genes (ZC03_051 to ZC03_055) are encoded by this region, and most of them were annotated as hypothetical. Weak hits suggest functions for the genes as shown in Table 5, but such evidence was not considered enough for a robust annotation. The only annotated gene in the cassette is a DrpA-like DNA recombination mediator. Other two indels events consist of unique sequences in ZC08 that encode one putative HNH homing endonuclease (ZC08_055) and three hypothetical proteins, respectively (Table 5). Details of the homing endonuclease insertion region will be discussed later in this work.

### tRNAs and codon bias

Most phages in the N4-like group present one to three tRNAs genes that are not encoded by the host genome. In this group, there are *Pseudomonas* phages without any tRNA genes while some *Salmonella* phages harbor 10 or more genes for several tRNAs [26]. In this regard, phages ZC03 and ZC08 are the first *Pseudomonas* N4-related phages to carry tRNAs genes. Analysis of the ZC03 and ZC08 tRNA genes revealed anti-codons for seven different amino acids, with proline and leucine present twice. The prediction software could not accurately assign the anti-codon for one ZC03 tRNA gene, which also happens to be the one missing in ZC08 genome. It seems that the equivalent region to this tRNA gene in ZC03 and ZC08 genomes may have accumulated enough substitutions to produce a pseudo tRNA gene (see Additional file 5: Figure S1).

We analyzed codon usage for phage ZC03 and the host *P. aeruginosa* PA14 proteins. Table 6 shows predicted codons concerning ZC03 and ZC08 tRNAs and usage bias for each codon among all the codons for the same amino acid. The results show that tRNAs carried by the phage correspond to codons rarely used by the host. There is even an extreme case for the codon UUA (Leu) whose tRNA is not encoded by *P. aeruginosa* PA14 genome. This data corroborates reports in the literature, suggesting that a selective recruitment of tRNAs compensates for the compositional differences between phage and host genomes [28]. The only exceptions to this pattern seem to be the tRNAs for asparagine and methionine, which do not present any detectable bias.

**Table 6:** Codon usage for phage ZC03 and *P. aeruginosa* PA14

Codon	UUA	CUA	AUG	AAC	CCA	AGA	UCA	ACA	GUA
Amino Acid	Leu	Leu	Met	Asn	Pro	Arg	Ser	Thr	Val
tRNAs encoded by phage genome	1	1	1	1	2	1	1	1	1^a^
tRNAs encoded by host genome	0	1	4	2	1	1	1	1	2
Random usage	0.166	0.166	1	0.5	0.25	0.166	0.166	0.25	0.25
Phage ZC03 usage	0.132	0.15	1	0.532	0.3	0.242	0.154	0.275	0.3
*P. aeruginosa* PA14 usage	0.003	0.013	1	0.85	0.047	0.007	0.014	0.025	0.059

^a^The anti-codon for one tRNA could not accurately be predicted, and, therefore, this might be a tRNA^Val^ pseudogene

We have investigated proteins with high frequency of proline/leucine and proteins with high usage of the nine codons corresponding to the tRNAs carried by the phages. These were an Rz lipoprotein (ZC03_025), a putative class II holin (ZC03_027), two putative homing endonucleases (ZC03_062 and ZC08_055), one hypothetical protein containing a cellulase-like domain (ZC03_047), plus some hypothetical proteins (for a complete list of genes and codon composition, see Additional file 6: Table S4). Some studies have shown that highly translated mRNAs encoding important proteins to the organism are less susceptible to codon negative bias and wobble base-pairing, since the translation on those cases could be less efficient [29, 30]. In this context, the presence of tRNA genes may be related with phage virulence to ensure optional translation of late genes and faster lytic cycle, as previously suggested in [28]. Thus, it seems that the genes listed above might be especially important for phage lytic activity. By all means, a more detailed investigation is necessary to corroborate the linkage between presence of tRNA genes in phage genomes and their virulence.

### Homing endonucleases insertion region

Homing Endonucleases (HEs) are site-specific DNA endonucleases encoded by genes inside mobile elements such as self-splicing introns and inteins (auto-processing protein domains). These mobile elements can insert themselves within conserved genes without altering their function due to their posterior self-splicing activity at the RNA or protein level [31]. They undergo a life cycle that starts with the invasion of a population, continues with the spreading through individuals, and ends when the element is fixed and is no longer under positive selection. At this point, the homing endonuclease gene (HEG) sequence degenerates and loses its function through random processes [32].

HEs are commonly found in phage genomes, with reports indicating up to 15 genes in phage T4 [33]. Although, phage T4 is thought to be an outlier, since many T4-like viruses have been studied and they do not have as many HEs. Moreover, it remains a challenge to understand the influence of HEs in producing phenotypes and in the mosaic evolution of phage genomes [34].

**Fig. 3:**
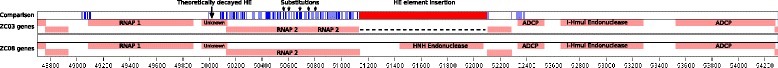
HNH endonuclease insertion region. Comparison of the HNH endonuclease insertion region in ZC03 and ZC08 genomes. Blue lines in the top strand are substitutions, the red block represents the HE element insertion in ZC08 only and blank spaces are identical aligned regions. ADCP: ATPase Domain Containing Protein

At least two different HEGs were identified within phages ZC03 and ZC08 genomes, both resembling endonucleases from the HNH family [31]. The homing endonucleases insertion region and HE-containing genes in phages ZC03 and ZC08 are listed in the Additional file 7: Table S5. Figure 3 shows a genome plot of this region, where one can observe a common element equally inserted within an ATPase-domain-containing protein (ADCP) of 350 aa in ZC03 and ZC08 genomes. Because of the insertion, this protein was predicted as two separated pieces, and multiple alignment of orthologous proteins in the *N4-like* cluster (VOCs ID: 18481) indicates that ZC03 and ZC08 are the only genomes to present an HE within this ORF. Searches of the fusion protein against the Reference Proteome HMMER database of HMMs [35] suggest that the protein is a ribonucleotide reductase. Similar cases of HE disrupting conserved ribonucleotide reductase genes were reported for phage Aeh1 and Twort [36, 37].

The second HE is inserted within the RNA polymerase (RNAP) subunit 2 gene in ZC08 genome only (Fig. 3). ZC08 specific HE was assigned to the ortholog group VOCs ID:18862, which also contains two other proteins from phages G7C (YP_004782150.1) and EE36P1 (YP_002898939.1). However, this homolog HE element is inserted in a different location inside phages G7C and EE36P1 genomes, more specifically between the genes RNAP1 and RNAP2 (YP_004782141.1 and YP_004782143.1 in phage G7C, respectively), which encode a two-subunits RNAP.

A closer investigation about the RNAP genes in phages from the *Podoviridae* family shows three organization types: (I) One single-unit protein of about 880 aa (T7-type), (II) two adjacent genes encoding for a two-subunits RNAP (N4-type), and (III) two genes spaced by one or more non-related canonical genes encoding a two-subunits RNAP (G7C-type). Multiple alignment of the RNAP protein sequences from T7-type, N4-type and G7C-type strongly suggests that the two subunits are actually non-overlapping pieces from the larger T7-type RNAP (see Additional file 8 for the alignment). Thus, the HE insertion within the RNAP2 gene in ZC08 genome may provide an insight to understand the evolutionary history of RNA polymerases in phages.

Altogether these findings suggest that single-unit and two-subunits RNAPs in *Podoviridae* may be linked by a common evolutionary pathway, as previously suggested in [38]. Our hypothesis is that a single-unit RNAP was present in the common ancestor of T7-like and N4-like phages. After these two lineages diverged, this single-unit protein was probably disrupted by the insertion of an HE element in the lineage that originated the N4-like phages. Since then, random events have led to two-subunits RNAP genes that continue to present affinity to assemble the complex required for transcription, as was experimentally demonstrated [38]. Although this hypothesis needs more supporting data, it is also corroborated by the comparative analysis of phage genomes from the T7-like and N4-like groups. Sequence inspection shows size variations in the spacing region between RNAP1 and RNAP2 genes. For instance, phage G7C presents only one putative HNH endonuclease (YP_004782142.1) between the RNAP subunits genes, while phage EE36P1 presents one putative HNH endonuclease (YP_002898939.1) plus seven predicted hypothetical proteins. There are examples from zero up to eight spacing genes between the RNAP1 and RNAP2 genes in N4-like phages.

### Phylogenetic analyses

**Fig. 4:**
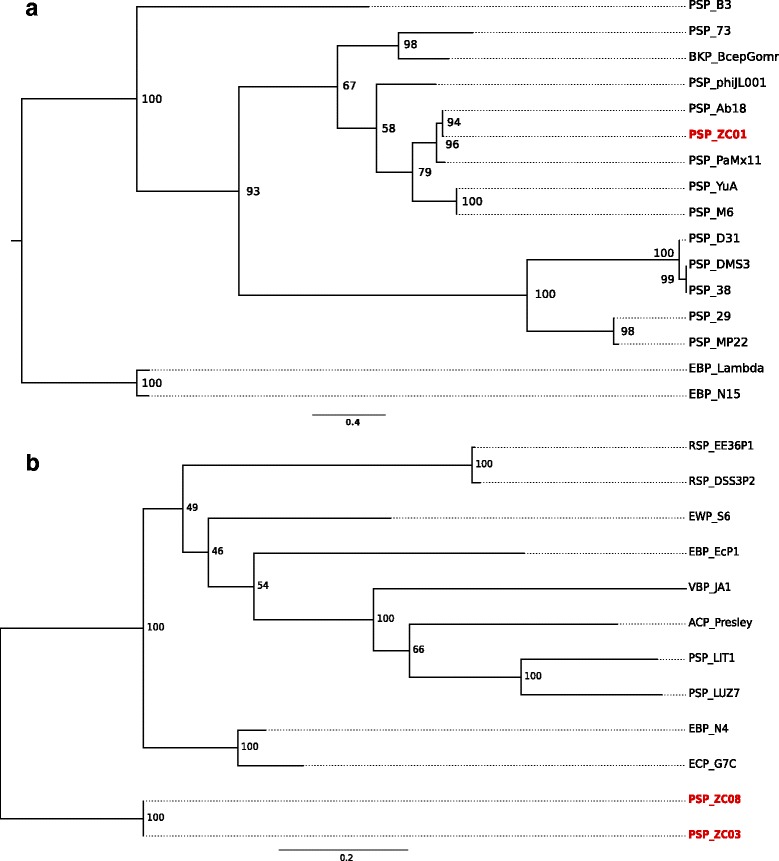
YuA-like and N4-like phylogenetic trees. **a** Maximum likelihood phylogenetic tree based on the *terL* gene for phages in the YuA-like VOCs cluster. The tree was rooted by two external groups represented here by Enterobacteria phage Lambda (EBP-Lambda) and Enterobacteria phage N15 (EBP-N15). **b** Maximum likelihood unrooted phylogenetic tree based on the *terL* gene for phages in the N4-like VOCs cluster. Bootstrap values are shown close to the nodes in percentages

We performed phylogenetic analyses based on the Terminase Large Subunit gene (*terL*) of each cluster of phages. The YuA-like cluster presents three different ortholog groups for this gene (VOCs ID:18306, 19671, 19804) which may indicate non-orthologous or distant orthologous proteins. A maximum likelihood phylogenetic tree was generated (Fig. 4a). The presence of three distinct groups, which correspond exactly to the VOCs ortholog groups assignment, suggests that these sequences have long undergone separate evolutionary pathways. Phages Lambda and N15 belong to an external group, since they are phages from the *Siphoviridae* family but from a different and closely related group (*Lambda-like* phages).

All *terL* genes from the N4-like cluster were assigned to the same ortholog group (VOCs ID: 18525), which was also part of the *N4-like* core-genome. Fig. 4b shows an unrooted maximum likelihood tree for phages from the N4-like group. Internal nodes display weak bootstrap support, indicating that the relationships among phages inside the N4-like cluster are unresolved. However, the bootstrap values strongly support the existence of two clades, one grouping ZC03 and ZC08 and another grouping *N4-like* and related phages. This data supports the claim that phages ZC03 and ZC08 constitute a new genus inside the *Podoviridae* family.

It is worth mentioning that building phylogenetic trees for phages remains a tough challenge due to the non-existence of marker genes present in all species. The *terL* gene represents a candidate for this purpose in the order *Caudovirales*, but it is clearly limited by its lack of identifiable homology among families inside the order.

### Host range and phage morphology

As previously mentioned, the three phages under study were isolated from composting samples using *P. aeruginosa* PA14 as host. In infection assays of *P. aeruginosa* PA14, phage ZC01 exhibits large lysis plaques with well-defined borders and diameter of 2.0-2.5 mm. ZC03 and ZC08 present much smaller lysis plaques (0.5-1 mm) than ZC01 (see Additional file 9: Figure S2). The three phages formed clear plaques, which is typical for lytic (virulent) phages. Given that ZC03/ZC08 probably belong to a new genus in the *Podoviridae* family, we performed one-step growth curve experiments for ZC03 as its archetype. The curve revealed a latent period of ~50 min with the number of phage particles reaching a peak at 240 min after infection and a calculated burst size of 10 phage particles per infected cell (see Additional file 10: Figure S3).

Phage host range was evaluated using 30 different strains, including bacteria from well-studied genera (e.g. *Escherichia*, *Enterococcus, Bacillus*), as well as several clinical *P. aeruginosa* isolates besides the reference strains PA14 and PAO1. Out of these, only four *P. aeruginosa* isolates were susceptible to phage lysis (Table 7). Higher lysis efficiency was observed only for strains PA14 and H6044, where clear plaques appeared even in more diluted titers for all the three phages. PAO1 strain was not susceptible to lysis. Additional file 11: Figure S4 shows images of the drop test for *P. aeruginosa* PA14 and PAO1 reference strains. These results indicate that the new phages present a narrow host range for *P. aeruginosa* strains*,* but other yet-to-be discovered potential hosts cannot be ruled out.

**Table 7:** Assessment of ZC01, ZC03 and ZC08 host range

Species/strain	Phage		
	ZC01	ZC03	ZC08
*Bacillus subtilis PY79*	-	-	-
*Chromobacterium violaceum* ATCC 124721	-	-	-
*Chromobacterium violaceum* isolated from Rio Negro	-	-	-
*Escherichia coli* MG1655	-	-	-
*Enterococcus faecalis* ATCC 29212	-	-	-
*Klebsiella pneumoniae* ATCC 13883	-	-	-
*Pseudomonas aeruginosa* PA14	C	C	C
*Pseudomonas aeruginosa* PAO1	-	-	-
*Pseudomonas aeruginosa* 442	-	-	-
*Pseudomonas aeruginosa* U456	-	-	-
*Pseudomonas aeruginosa* H6086	C	-	-
*Pseudomonas aeruginosa* 95291	-	-	-
*Pseudomonas aeruginosa* 5172	-	-	-
*Pseudomonas aeruginosa* U3554	-	-	-
*Pseudomonas aeruginosa* H6044	C	C	C
*Pseudomonas aeruginosa* 5757	T	T	-
*Pseudomonas aeruginosa* 5728	-	-	-
*Pseudomonas aeruginosa* 5031	-	-	-
*Pseudomonas aeruginosa* 438	-	-	-
*Pseudomonas aeruginosa* 426C	-	-	-
*Pseudomonas aeruginosa* 5728NF	-	-	-
*Pseudomonas aeruginosa* 5833	-	-	-
*Pseudomonas aeruginosa* U514	-	-	-
*Pseudomonas aeruginosa* PHB64	-	-	-
*Pseudomonas aeruginosa* DE01	-	-	-
*Pseudomonas aeruginosa* 48.1997A	-	-	-
*Serratia marcescens* isolated from Rio Negro	-	-	-
*Staphylococcus aureus* ATCC 29213	-	-	-
*Stenotrophomonas maltophilia* ATCC 13637	-	-	-
*Xanthomonas axonopodis pv. citri 306*	-	-	-

C (clear phage plaque); T (turbid phage plaque); - (no phage plaque). See Additional file 12: Table S6 for references of these strains

**Fig. 5:**
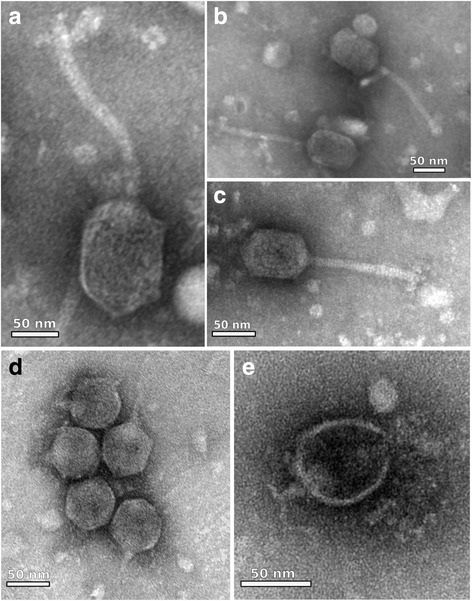
Electron micrographs of phages ZC01, ZC03 and ZC08. Transmission electron micrographs of negatively stained *Pseudomonas* phages virions found in composting: **a**–**c** *Pseudomonas* phage ZC01 with typical morphology of members of the *Siphoviridae* family; (**d**, **e**) *Pseudomonas* phages ZC03 and ZC08, full virions and empty shelled, respectively, with typical morphology of members of the *Podoviridae* family. Note the short tail

Morphology features for the three new phages virions were assessed by transmission electron microscopy. Phage ZC01 has the typical morphology for phages of the *Siphoviridae* family and more specifically for phages of the *YuA-like* group [16] (Fig. 5a–c). We identified a prolate and more elongated head of ~80 nm by ~58 nm (morphotype B2). Tail is ~150 nm long, cross-banded, flexible and non-contractile, with a terminal structure resembling short fibers. As predicted from genomic comparisons, phages ZC03 and ZC08 belongs to *Podoviridae* and as such, exhibit morphological characteristics of phages from this family (Fig. 5 d and e, respectively). The electron micrographs show their icosahedral head of ~72 nm by ~59 nm and a short tail ~21 nm long with terminal fibers.

### Putative cell lysis associated proteins

We have screened phage genomes for genes possibly involved in pathways of cell lysis and biomass degradation, since the lysis and turnover of bacterial cell components constitute important steps in the process of nutrients recycling [39]. Most genes involved in cell lysis and biomass degradation in phages are responsible for breaking cell wall peptidoglycan components and making pores in the lipid membrane, which are important steps in infection or in the release of phage progeny [40]. Nine proteins were found, including peptidoglycan hydrolases, N-acetylmuramidases, Rz lipoproteins, holins and an endolysin (Table 8). For example, a peptidoglycan hydrolase gp181-like (831 aa) is present in both ZC03 and ZC08 genomes. These proteins were assigned to the ortholog group VOCs ID: 18902, which contains only these two genes and no other homologs in the N4-like cluster. We identified a central lysozyme-like domain (pfam1464) and N-acetyl-D-glucosamine binding sites in the protein. Previous reports indicate a similar architecture for *Pseudomonas* phage phiKZ gp181 (Uniprot Q8SCY1) [41].

**Table 8:** Putative cell lysis associated proteins encoded by phages ZC01, ZC03 and ZC08

Phage	Gene name	Annotation	Size (aa)	Additional information
Phage ZC01	ZC01_075	Putative endolysin	168	TIGR02594 family protein
Phage ZC01	ZC01_076	Putative holin	70	Two transmembrane domains found with TMHMM
Phage ZC01	ZC01_078	Rz/Rz1 lipoprotein	182	Bacteriophage Rz lysis protein (pfam03245)
Phage ZC03	ZC03_016	Peptidoglycan hydrolase gp181-like	831	N-acetyl-D-glucosamine binding site; lysozyme-like domain (pfam01464)
Phage ZC03	ZC03_025	Putative RZ/Rz1 lipoprotein	164	Similar to Rz/RzI spanin protein in phage EC1-UPM (AGC31575.1)
Phage ZC03	ZC03_026	N-acetylmuramidase	194	Glycosyl hydrolase 108 (pfam05838); Pepitidoglycan binding domain
Phage ZC08	ZC08_016	Peptidoglycan hydrolase gp181-like	831	N-acetyl-D-glucosamine binding site; lysozyme-like domain (pfam01464)
Phage ZC08	ZC08_025	Putative Rz/Rz1 lipoprotein	164	Similar to Rz/RzI spanin protein in phage EC1-UPM (AGC31575.1)
Phage ZC08	ZC08_026	N-acetylmuramidase	194	Glycosyl hydrolase 108 (pfam05838); Pepitidoglycan binding domain

Additional information include significant predictions of binding sites, transmembrane regions, domain regions and significant hits to similar proteins

### *Pseudomonas aeruginosa* biofilm degradation

**Fig. 6:**
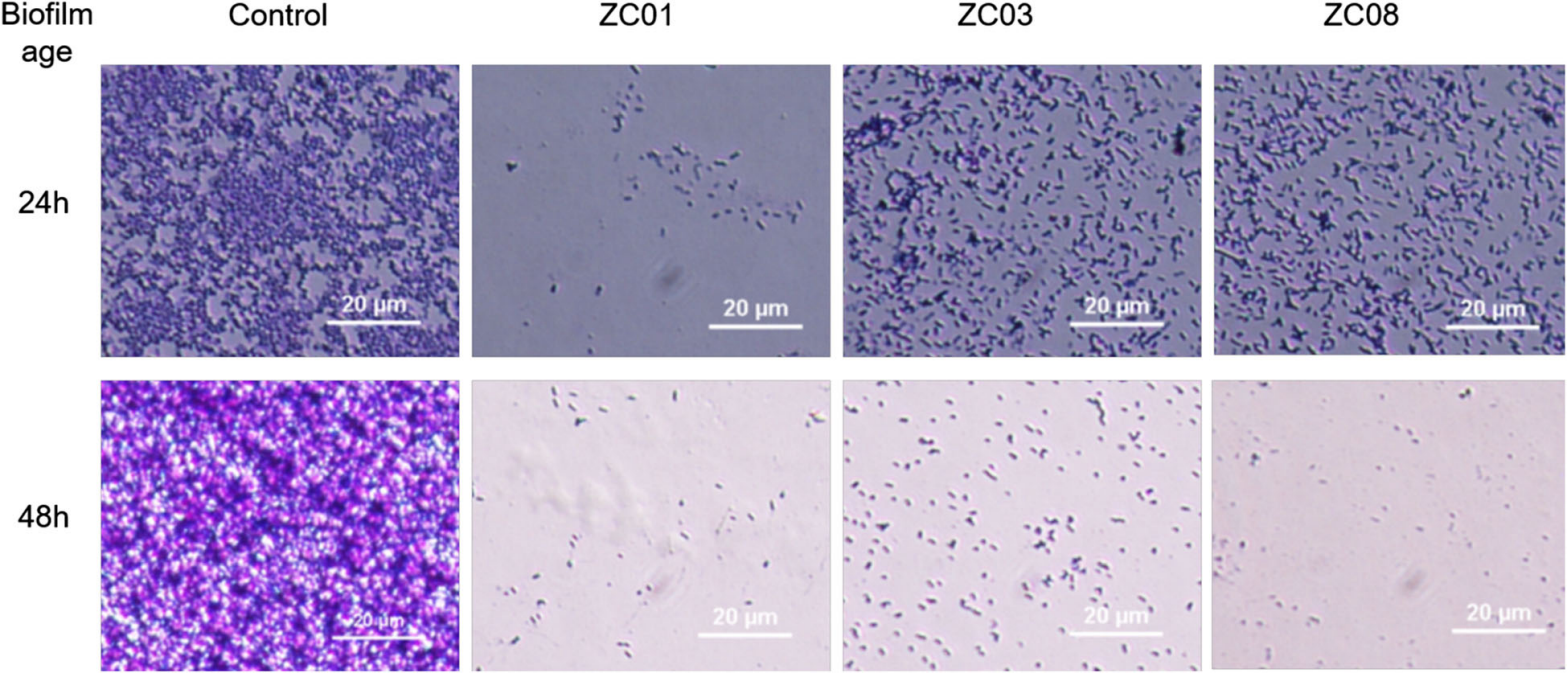
Biofilm degradation assay. Biofilms of 24 h and 48 h were exposed to phages ZC01, ZC03, and ZC08 for 24 h. Image shows the results after exposure for each of the phages compared to the control, which was exposed to a buffer solution only. Images are representative of n=4 replicates

To investigate the ability of phages to mediate biofilm degradation, we challenged 24/48 hours *P. aeruginosa* PA14 biofilms with the three different phages isolated in this study. Exposure to the three phages strongly reduced biofilm cell densities, mainly for phage ZC01 (Fig. 6). These results indicate a promising degradation potential for these three new phages against *P. aeruginosa* PA14 biofilms. This strain is highly virulent in susceptible animal hosts and known to form a biofilm structure resistant to currently available antibiotics [42]. In these assays we used lower phage titers (~5 × 10^5^ PFU ml^-1^) than the titers normally used in biofilm degradation assays (1 × 10^6^ PFU ml^-1^ to × 10^10^ PFU ml^-1^) [43–45] highlighting the antibiofilm effectiveness of these phages.

### Phages in action: metagenomic and metatranscriptomic analyses in the composting process

**Fig. 7:**
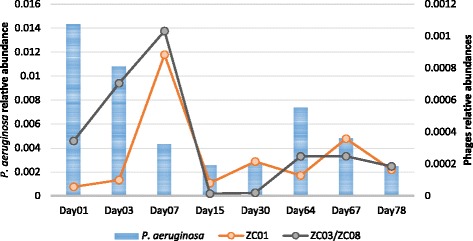
Phage-Host relative abundance in the composting metagenome. Relative abundance of metagenomic reads through the composting process. Raw reads count for phages and host was divided by the total number of reads in each sample and normalized by the genome size of the organism (given in percentage)

This work is part of a project that aims to understand the composting process at the microbial and molecular levels [7, 9]. In this project, time-series samples of a composting unit were obtained and corresponding DNA and mRNA sequence datasets were generated. As the samples from this composting unit were used for both the metagenomic and phage isolation studies, it was feasible to verify the presence of phages/host in the DNA and mRNA sequence datasets for each sampling day. We indeed found sequences that correspond to these genomes in all datasets and the relative abundance was inferred for phage-host populations (Fig. 7). Phages ZC03 and ZC08 relative abundance variation parallels that of *P. aeruginosa* but with an apparent delay, which is consistent with mathematical models that have been proposed for phage-host variation [46, 47]. We calculated a correlation score applying the Local Similarity Analysis (LSA) technique for time-series samples [48], and a positive LS score of 0.71 was obtained for *P. aeruginosa* and phages ZC03/ZC08 abundances (*p*-value < 0.02). This data suggests that, in this environment, *P. aeruginosa* and phages ZC03/ZC08 may present a mutualistic relationship that is characteristic of lysogenic phages, as discussed in [49]. We emphasize that our experimental results show that ZC01, ZC03 and ZC08 are lytic phages in the conditions we used for cultivation in PA14. Additionally, we also performed lysogeny experiments and the results showed negligible frequency of lysogeny (<1%) for the three phages. However, we cannot rule out the possibility that these phages could establish a mutualistic relationship with their host in different environmental conditions.

**Fig. 8:**
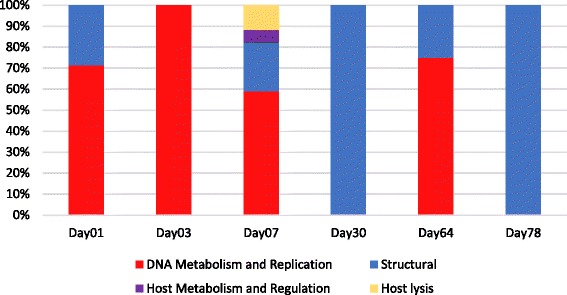
Phage-related functions in the composting microbial community. Phage-related protein functions through the composting process assessed by the identification of mRNA sequences from phages and the host *P. aeruginosa* in time-series metatranscriptomic samples. Values are shown in percentage of the total of proteins with known function

We investigated active phage-related functions in the composting process through the identification of metatranscriptomic reads mapped to phage genes. Fig. 8 shows the proportion of mRNA reads identified for each day in the respective function. “Structural” and “DNA metabolism and replication” are the predominant phage functions expressed through the days of the composting process. We identified mRNA for host lysis in the sample of day 7, more specifically mRNA reads for a class II phage holin (ZC03_027). It is interesting to note that a spike in phage abundance is also observed on day 7, as well as a marked decrease in host abundance (Fig. 7). This observation suggests a cause-effect relationship, but additional studies are necessary to gather additional evidence for this hypothesis.

## Conclusions

In this work, three new *Pseudomonas* phages have been characterized in terms of genomic structure, genes, and the putative proteins encoded by their genomes. Two of the three phages present remarkable novelty at the genomic level and may be members of a new genus in the *Podoviridae* family. Comprehensive comparative analyses of the new phages in a context with phages from YuA-like and N4-like clusters provided insights about the evolution and diversity of tailed phages. Moreover, infectivity and biofilm degradation experiments suggest a narrow host range and a potential as anti-microbial agents against *P. aeruginosa* PA14 infections, warranting further studies to explore this promising application. Finally, metagenomic and metatranscriptomic analyses provided data to situate phages ZC01, ZC03, and ZC08 in the microbial community to which they belong, yielding interesting clues about phage population dynamics and phage transcript presence in this complex environment.

## Methods

### Bacterial strains and growth conditions

*Pseudomonas aeruginosa* PA14 cells were grown at 37 °C in LB-medium. Solid LB medium contained 1.5% (w/v) of Bacto agar (Difco) and the soft agar top-layer contained 0.7% of Bacto agar. All strains were subcultured once and glycerol stocks were done and stored frozen at -80 °C until further use.

### Phages isolation and propagation

Composting sample for phage isolation was collected from the composting facility in the São Paulo Zoo Park, São Paulo, Brazil following the procedure previously described [9] upon 67 days after completion of the composting pile. The procedure for phage isolation was adapted from [50]. The compost sample (~75 g) was suspended in 300 mL of SM buffer (10 mM MgSO_4_; 50 mM Tris-HCl, pH 7.5) containing 3% NaCl (w/v), dispensed into 50 mL centrifuge tubes and incubated for 60 min at 4 °C. Suspensions were homogenized for 5 min at maximum speed using the Tissuelyser II (Qiagen) and centrifuged at 3000 xg for 10 min. The supernatants were filtered through a 0.2 μm membrane and immediately used for infection by *P. aeruginosa* PA14 using the soft-agar overlay method [51]. After overnight incubation at 37 °C, several individual lytic plaques were collected, suspended in 100 μL of SM buffer and used for a new round of infection to warrant phage purification. The genomes of seven phage isolates were fully sequenced and out of them, three were found to be distinct (ZC01, ZC03 and ZC08). The other five isolates were identical to one of these three selected phages.

Phages were propagated using the soft-agar overlay method [51] using *P. aeruginosa* PA14 as the host strain. Briefly, 10 μL of isolated phage lysate were mixed with overnight bacterial culture and 3-5 mL of top-agar LB, and then added onto a LB Petri dish. After incubation, the lysate from a clear Petri dish was eluted with SM buffer and stored at 4 °C for further use. High titer phage suspensions were prepared using CsCl gradient centrifugation using standard protocols.

### Phage titration and one-step growth curve

Bacteriophage titer was determined as described by [51]. Briefly, 100 μL of diluted phage suspension, 100 μL of a *P. aeruginosa* PA14 overnight culture, and 5 mL of LB top agar were mixed in a tube and poured into a LB agar-containing Petri dish. After incubation for 18 h at 37 °C, plaque forming units (PFU) were enumerated.

For one-step growth curve, a phage suspension was added to *P. aeruginosa PA14* culture at multiplicity of infection (MOI) of 0.01. After incubation at 37 °C for 10 min to allow phages adsorption, the mixture was centrifuged for 30 s at 12,000 xg. The supernatant was collected and further centrifuged for 2 min 12,000 xg for evaluation of the fraction of non-adsorbed phages. Pellet was resuspended in 30 mL of LB, incubated at 37 °C without shaking and 300 μL samples were collected every 10 min and diluted for PFU enumeration.

### Phage DNA extraction and Illumina MiSeq sequencing

For DNA extraction, phages were propagated in *P. aeruginosa* PA14 strain and collected after complete bacterial lysis, using 10 mL of SM buffer per Petri dish. The phage suspension was filtered through a 0.2 μm membrane and viral particles were precipitated with 10% polyethylene glycol (PEG) 8000 (w/v) and 1 M NaCl overnight at 4 °C. Viral particles were collected by centrifugation at 3,000 × g for 5 min. The pellet was suspended in 1 mL of SM buffer and treated with DNAse (TURBO DNA-free, Life Technologies) as a way attempt to reduce contamination with *P. aeruginosa* DNA. The intact viral particles suspension were treated with phenol:chloroform:isoamylalchool and phage DNA was extracted using MoBio PowerMax Soil DNA kit (MoBio Laboratories). Purified phage DNA was subjected to a final clean-up step using QIAamp mini spin columns (Qiagen, USA) and stored at -80 °C.

DNA purity and concentration were evaluated on a ND-1000 spectrophotometer (Nano Drop Technologies, USA) at 260 nm, 280 nm and 230 nm. Further quantification was performed with Quant-iT Picogreen dsDNA assay kit (Life Technologies, USA). DNA integrity was examined with DNA 7500 chip using 2100 Bioanalyzer and were mostly enriched in fragments higher than 10 kbp. Shotgun genomic libraries were prepared using an Illumina Nextera DNA library preparation kit (Illumina, Inc., USA) with total DNA input of 20-35 ng. The resulting DNA fragment libraries were cleaned up with Agencourt AMPure XP beads (Beckman Coulter, Inc., USA) and fragment size within the range of 400-700 bp was verified by running in the 2100 Bioanalyzer using Agilent High Sensitivity DNA chip. Quantification of Illumina sequencing libraries with KAPA Library Quantification Kit, normalization, and pooling were performed following standard protocols for sequencing in the Illumina MiSeq platform. Pooled libraries were subjected to one run using the MiSeq Reagent kit v2 (500-cycle format, paired-end (PE) reads). On average, Illumina PE read1 and read2 presented, respectively, >80% and >75% of bases with quality score at least 30 (Q30).

### Genome assembly

Raw reads were subject to host DNA contamination removal with Deconseq [52] followed by a three-way protocol for digital normalization of high coverage libraries using KHMER Perl scripts [53]. Resulting reads were assembled with MIRA 4 (mode: genome, accurate, others parameters default) [54] and final genomes were assessed by manual inspection of coverage and mapping on IGV [55].

### Clusters of phages

In order to define clusters of similar genomes to be implemented in the VOCs database, we counted the number of shared genes between two phage genomes according to Phage Ortholog Groups (POGs) data available by FTP [56]. Then, numbers of shared genes were used to calculate the *Jaccard index* (or Jaccard coefficient of similarity) for each pair of genomes according with the following expression:

1 $$ \begin{array}{l} J\left( A, B\right)=\frac{\left| A{\displaystyle \cap } B\right|}{\left| A{\displaystyle \cup } B\right|}=\frac{\left| A{\displaystyle \cap } B\right|}{\left| A\left|+\right| B\left|-\right| A{\displaystyle \cap } B\right|}\kern.5em \\ {}0\le J\left( A, B\right)\le 1\kern1em \end{array} $$

Where A and B are the set of genes from A and B, respectively.

We consider that this index reflects similarity with more reliability than only using an absolute number of shared genes, since the J-index also consider the similarity between the total number of genes in the two phages being assessed. Lastly, we selected one reference genome for each one of the clusters and grouped genomes with a pairwise *J*(*ref*, *X*)  ≥ 0.1.. Phages YuA and N4 were chosen as reference genomes due to their close relationship (attested by BLASTN searches to the nt NCBI database in Jan 2016) with ZC01 and ZC03/ZC08, respectively. The J-index cutoff was defined based on exploratory analyses of our data.

Ortholog assignment methodology and VOCs implementation were made as previously described in [21]. All VOCs data and genomic information about phage genomes in the two clusters used in this work are public available through a Java client in the VBRC web platform [22].

### Genomic and functional characterization

Genes were predicted by GenMarkS [57] and Prodigal [58] using models for phage genes and t-RNA predictions were performed by Aragorn [59]. Proteins were automatically annotated by ProKKA [60] followed by additional manual characterization using CDD-Search [61], HMMER-Search [35] and BLAST searches [23] (Jan 2016). Hits were considered robust and significant for annotation when above the following alignment thresholds: E-value: 10E-5, alignment coverage: 60% and identity: 50%. VOCs ortholog groups and embedded tools were used for transitive annotation and comparative analyses [21, 62, 63].

### Phylogenetic analyses

We identified ortholog groups for the Terminase Large Subunit gene (*terL*) by similarity in each of the clusters and performed multiple alignments through the VOCs GUI interface using MAFFT 7 (L-INS-i iterative algorithm and others parameters default) [64]. Guidance [65] was used to test the robustness of the multiple alignments and columns with confidence score below 0.4 were removed. The evolutionary history was inferred by using the maximum likelihood method based on the Whelan-Goldman model [66] and Le-Gascuel model [67] for the *Siphoviridae* cluster and *Podoviridae* cluster, respectively. Discrete Gamma distributions with 5 categories were used to model evolutionary rate differences among sites. Robustness of branches were tested by 1000 interactions of bootstrap [68]. Best fitting models and evolutionary analyses were conducted in RAxML version 8 [69].

### Analyses of phages abundance in composting samples

Metagenomics and metatranscriptomics datasets from composting time-series used in the analyses were generated from a composting unit at the Sao Paulo Zoo Park (Brazil) and are publicly available in MG-RAST (see [7] for sampling details and accession numbers). Sequences were subject to mapping using Bowtie2 [70] (default parameters) against the isolated phage genomes and *P. aeruginosa PA14*. Reads mapping were considered as belonging to the new phages or the host and counted. Relative abundances were calculated dividing the number of reads by the total number of sequencing reads generated for the sample being analyzed. We applied genome size normalization in order to compare relative abundance of phages and the host *P. aeruginosa*.

### Phage host range assay

Host range of the isolated phages was assessed by drop test against 30 bacterial strains (Additional file 12: Table S6), including the reference *P. aeruginosa* strains PA14 and PAO1. Bacterial lawns of the different strains were propagated in LB agar plates by plating 100 μL of overnight cultures and 10 μL drops of phages suspension at 10^7^, 10^8^, 10^9^ and 10^10^ PFU mL^-1^. The plates were incubated for 18 h and then checked for presence of lysis plaques.

### Lysogeny assay

Phage suspensions (100 μL) diluted to 10^10^ PFU mL^-1^ were seeded in LB plates. Overnight culture of *P. aeruginosa* PA14 at OD_600nm_ = 1.0 was serially diluted (10^-4^ to 10^-7^) and 100 μL of each dilution were mixed with 4.5 mL LB top agar and added to phage seeded plates. Plates were incubated at 37 ° C for 3 days for CFU (colony forming unit) enumeration. Unseeded plates were used as control.

### Study of bacteriophages effects on biofilm formation

Biofilms were allowed to form on 8-well chamber stainless slides for 24 h or 48 h as described in [71]. Briefly, bacterial culture (200 μl) with an OD_600_ of 0.05 - 0.1, which corresponds to approximately 1-2 × 10^7^ cells was added to each well. The slide was incubated at 37 °C for 24 h or 48 h without shaking. The medium was replaced once a day during the whole experiment. Afterwards, the slides were washed twice with LB medium and the biofilms were challenged with 100 μl of LB and 100 μl of phage solution with a concentration of 5 x 10^5^ PFU ml^-1^ during 24 h at 37 °C. Control experiments were performed at the same conditions with the slides incubated with 100 μl of LB and 100 μl of SM buffer. Biofilms attached to slides before and after phage infection were stained with 1% of crystal violet solution in ethanol 96% and analyzed in the microscope.

### Transmission electron microscopy

For transmission electron microscopy, copper grids covered with carbon-coated Formvar films were floated, membrane side down, on a drop of phage suspension for about 10 min. After eliminating excess liquid and washing with distilled water, grids were floated on a drop of 1% (w/v) uranyl acetate for 5 min. After eliminating excess liquid, dried grids were examined in a JEOL JEM 1011 or Philips EM 300 transmission electron microscope and the images registered digitally. At least 10 virions were examined for each phage preparation.

## Additional files

Additional file 1: Table S1. Assembly details and benchmarks for phages ZC01, ZC03 and ZC08 genomes. (XLSX 9 kb)

Additional file 2: tblastn search output from ZC01 Rz1 lipoprotein against the YuA-like cluster of genomes. (TXT 4 kb)

Additional file 3: Table S2. Detailed information about ZC01 annotation. (XLSX 38 kb)

Additional file 4: Table S3. Detailed information about ZC03 and ZC08 annotation. (XLSX 45 kb)

Additional file 5: Figure S1. tRNA genes in ZC03 and ZC08 genomes. (PDF 21 kb)

Additional file 6: Table S4. ZC03 and ZC08 genes list and codon usage for amino acids of interest. (XLSX 28 kb)

Additional file 7: Table S5. List of the genes present in the Homing endonuclease insertion region for phages ZC03 and ZC08. A list of gene features is also presented. (XLSX 11 kb)

Additional file 8: Multiple sequence alignment of the T7-like RNA polymerase and N4-like RNA polymerases subunits 1 and 2. (TXT 12 kb)

Additional file 9: Figure S2. Phages ZC01, ZC03 and ZC08 lysis plaques morphology. (PDF 127 kb)

Additional file 10: Figure S3. One-step growth curve for phage ZC03. (PDF 88 kb)

Additional file 11: Figure S4. Phage drop test for *P. aeruginosa* PA14 and PAO1 reference strains. (PDF 260 kb)

Additional file 12: Table S6. Source of strains used in host range assays shown in Table 7. (XLSX 13 kb)

## Abbreviations

ADCP: ATPase domain containing Protein
HE: Homing Endonuclease
HEG: Homing endonuclease gene
ICTV: International Committee for Taxonomy of Viruses
NCBI: National Center for Biotechnology Information
OG: Ortholog group
POGs: Phage Ortholog Groups
RNAP: RNA Polymerase
*terL*: Terminase Large Subunit
VOCs: Viral Orthologous Clusters
