# Supplementary material for: Three novel Pseudomonas phages isolated from composting provide insights into the evolution and diversity of tailed phages
Source: BMC Genomics. 2017 May 4;18:346. doi: 10.1186/s12864-017-3729-z (PMC5418858; doi:10.1186/s12864-017-3729-z)
Supplement: Supplementary file 5 — tRNA genes in ZC03 and ZC08 genomes. (PDF 21 kb) [file 12864_2017_3729_MOESM5_ESM.pdf]

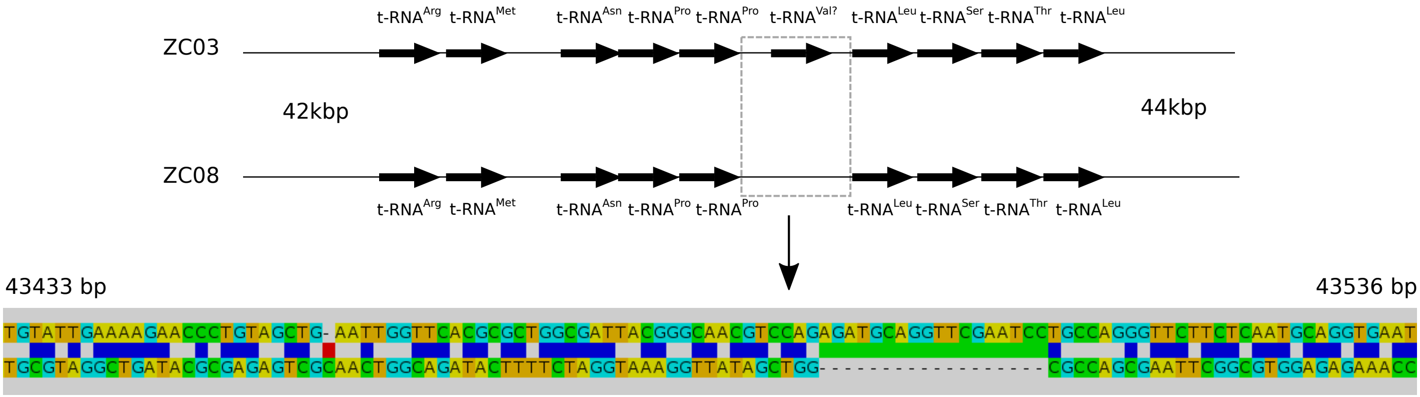

Figure S1: Genomic region with the tRNA genes in ZC03 and ZC08. Region corresponding to the sixth tRNA gene is highlighted due to the differences between the genomes; it may be a pseudogene.
