# Supplementary material for: Three novel Pseudomonas phages isolated from composting provide insights into the evolution and diversity of tailed phages
Source: BMC Genomics. 2017 May 4;18:346. doi: 10.1186/s12864-017-3729-z (PMC5418858; doi:10.1186/s12864-017-3729-z)
Supplement: Supplementary file 9 — Phages ZC01, ZC03 and ZC08 lysis plaques morphology. (PDF 127 kb) [file 12864_2017_3729_MOESM9_ESM.pdf]

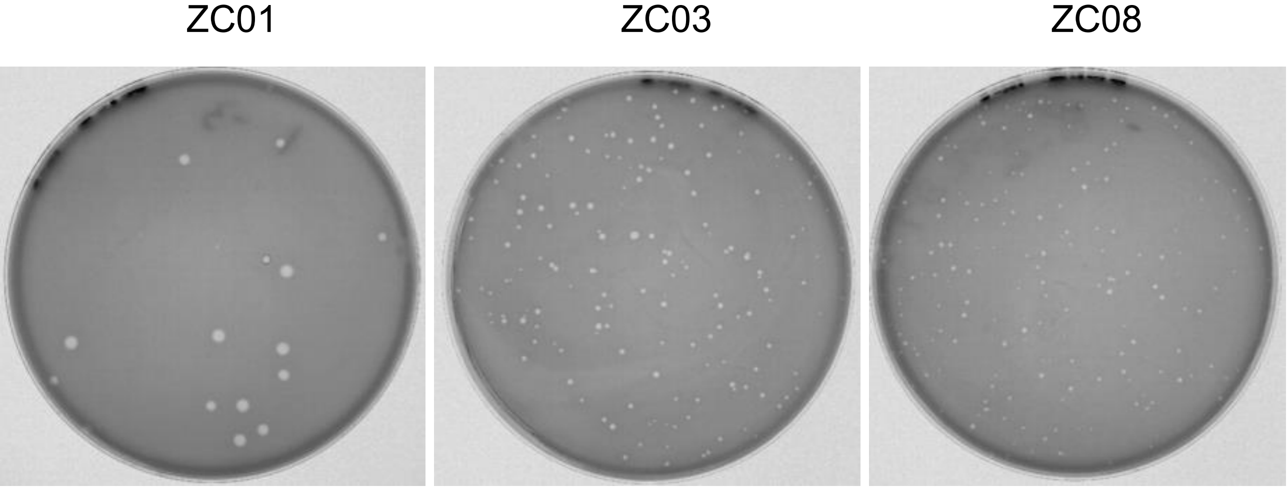

Figure S2. Lysis plaques formed on *P. aeruginosa* PA14 lawn. The same volume of a purified phage suspension and a bacterial overnight culture were mixed with LB top agar and plated in LB-agar. Plates were incubated for 18 h at 37º C after which plaque forming units (PFU) were checked.
