# Supplementary material for: Three novel Pseudomonas phages isolated from composting provide insights into the evolution and diversity of tailed phages
Source: BMC Genomics. 2017 May 4;18:346. doi: 10.1186/s12864-017-3729-z (PMC5418858; doi:10.1186/s12864-017-3729-z)
Supplement: Supplementary file 10 — One-step growth curve for phage ZC03. (PDF 88 kb) [file 12864_2017_3729_MOESM10_ESM.pdf]

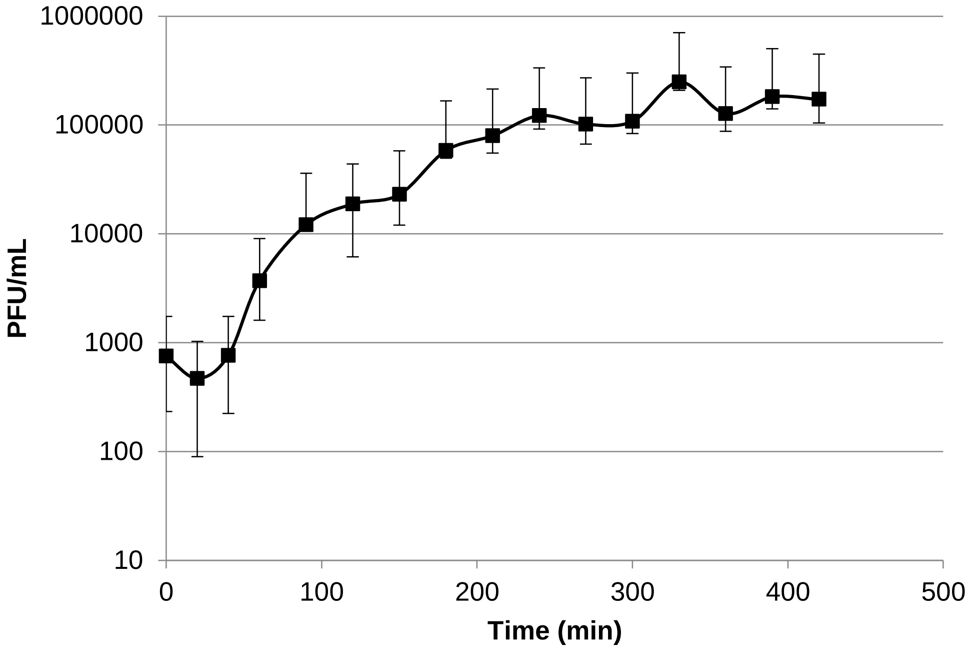

Figure S3: One-step growth curve for phage ZC03 showing a latent period of ~50 min. A plateau is reached at ~240 min and used to calculate the burst size. Bars indicate mean ± min/max of three independent experiments.
