# Supplementary material for: Three novel Pseudomonas phages isolated from composting provide insights into the evolution and diversity of tailed phages
Source: BMC Genomics. 2017 May 4;18:346. doi: 10.1186/s12864-017-3729-z (PMC5418858; doi:10.1186/s12864-017-3729-z)
Supplement: Supplementary file 11 — Phage drop test for P. aeruginosa PA14 and PAO1 reference strains. (PDF 260 kb) [file 12864_2017_3729_MOESM11_ESM.pdf]

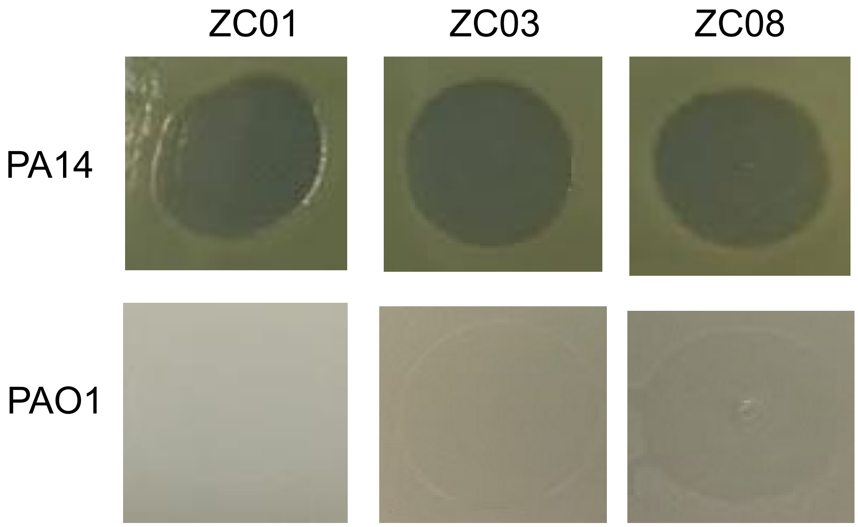

Figure S4: Phage drop test for *P. aeruginosa* PA14 and PAO1 reference strains. Bacterial lawns of *P. aeruginosa* PA14 and PAO1 strains were propagated in LB agar plates by plating 100 µL of overnight cultures and 10 µL droplets of phages (10^7 - 10^{10} PFU ml^{-1}). The plates were incubated 18 h at 37° C and checked for presence of lysis plaques. Only plaques formed with 10^{10} PFU ml^{-1} droplets are shown.
